# A whitefly effector Bsp9 targets host immunity regulator WRKY33 to promote performance

**DOI:** 10.1098/rstb.2018.0313

**Published:** 2019-01-14

**Authors:** Ning Wang, Pingzhi Zhao, Yonghuan Ma, Xiangmei Yao, Yanwei Sun, Xiande Huang, Jingjing Jin, Youjun Zhang, Changxiang Zhu, Rongxiang Fang, Jian Ye

**Affiliations:** 1 State Key Laboratory of Plant Genomics, Institute of Microbiology, Chinese Academy of Sciences, Beijing, People's Republic of China; 2 State Key Laboratory of Crop Biology, Shandong Key Laboratory of Crop Biology, Shandong Agricultural University, Taian, Shandong, People's Republic of China; 3 China Tobacco Gene Research Center, Zhengzhou Tobacco Research Institute of CNTC, Zhengzhou, Henan, People's Republic of China; 4 Institute of Vegetable and Flower, Chinese Academy of Agriculture Sciences, Beijing, People's Republic of China; 5 University of the Chinese Academy of Sciences, Beijing, People's Republic of China

**Keywords:** whitefly, TYLCV, salivary effector, WRKY33, innate immunity, Bsp9

## Abstract

Whiteflies, *Bemisia tabaci* (Hemiptera), are pests causing economic damage to many crops, capable of transmitting hundreds of plant vector-borne viruses. They are believed to secrete salivary protein effectors that can improve vector colonization and reproductive fitness in host plants. However, little is known about effector biology and the precise mechanism of action of whitefly effectors. Here, we report a functional screening of *B. tabaci* salivary effector proteins (Bsp) capable of modulating plant innate immunity triggered by plant endogenous pattern peptide Pep1. Four immunity suppressors and two elicitors were identified. Bsp9, the most effective immunity suppressor, was further identified to directly interact with an immunity regulator WRKY33. We provide evidence that Bsp9 may suppress plant immune signalling by interfering with the interaction between WRKY33 and a central regulator in the MAPK cascade. The interference by Bsp9 therefore reduces plant resistance to whitefly by inhibiting activation of WRKY33-regulated immunity-related genes. Further detailed analysis based on transgenic plants found that whitefly effector Bsp9 could promote whitefly preference and performance, increasing virus transmission. This study enriches our knowledge on insect effector biology.

This article is part of the theme issue ‘Biotic signalling sheds light on smart pest management’.

## 1. Introduction

Piercing/sucking feeding insects cause extensive crop losses directly [1]. Besides ingesting plant sap, phloem-feeding insects, such as whitefly, transmit 80% of known plant vector-borne pathogens and cause huge economic loss indirectly. During feeding, insects secrete salivary protein into plant cells together with virus particles. Although, for plant and animal microbes, it is well established that effectors target host proteins to manipulate host cell processes and promote infection and disease [2], understanding the effector biology of insects is still at a very early stage. So far, only a small number of salivary effectors from aphid species have been identified and a few of them have been confirmed by their functions in plant–insect interactions [3]. Among these insects with identified effectors, the broad host range aphid *Myzus persicae* is the most researched species. Some of effectors from *M. persicae* and homologues from other aphid species have been successfully identified through genomics. A few of them, such as Mp1 (PIntO1) and Mp2 (PIntO1), Mp10, Mp42, Mp55, Mp56, Mp57, Mp58 and others, have been functionally characterized to target host plant proteins to modulate immunity for enhancing aphid fitness [3–5]. However, the effectors from other insects, such as planthopper and whitefly, remain elusive.

Whitefly, *Bemisia tabaci* (Hemiptera), is a polyphagous insect and a supervector, transmitting more than 300 plant virus species, that is a threat for many crops across the globe [1]. Whitefly might mediate the suppression of plant defences by secreting protein effectors to improve host colonization and reproductive fitness [6,7]. The whitefly-transmitted monopartite geminivirus begomoviruses are frequently associated with pandemic crop diseases such as tomato yellow leaf curl virus (TYLCV) and tomato yellow leaf curl China virus [8]. We have shown that geminiviruses could repress defensive responses in infected plants leading to an improved fitness of their vector, whitefly, thereby promoting vector performance and, in turn, facilitating pathogen spread [9], but it is unknown whether the infestation of whitefly could affect plant immunity, possibly by secreting salivary effectors.

Plants fend off attacks from herbivores and pathogens in various ways, e.g. via physical barriers, volatile or non-volatile compounds, and through induction of defensive responses mainly controlled by phytohormones and innate immunities [10–12]. The phytohormone jasmonate (JA) is known to be indirectly manipulated by viruses to promote whitefly performance [9,13]. Plant innate immunity includes two major types of resistance mechanisms against pathogens and herbivores. The first layer is the pathogen-associated molecular pattern or damage-associated molecular pattern (DAMP)-triggered immunity, so-called pattern-triggered immunity (PTI). The second layer is a more specific effector-triggered immunity (ETI) [14–16]. PTI is a multistep response, which is triggered upon plant pattern recognition receptors recognizing the conserved pathogen molecules or endogenous peptide elicitors such as Pep1–Pep7 family [17,18]. PEPR1 and PEPR2 encode receptors to recognize Pep1 in *Arabidopsis*. The AtPep–PEPR system has been reported to be induced by *Spodoptera littoralis* feeding. Thus, Pep peptides function as DAMPs in response to wound- and herbivory-induced stresses [18–20]. Subsequently, many downstream signalling events are initiated, including activation of the mitogen-activated protein kinase (MAPK) cascades and the transcription of defensive genes, especially anti-pest defensin gene (*PDF1.2*) in *Arabidopsis thaliana* [21–25].

Whiteflies are important agricultural pests, but little is known about their effector biology. The goal of this study was to establish an efficient functional screening system of whitefly effectors and to elucidate the precise mechanism of whitefly effectors in the interaction with host and virus. Here, we identify a whitefly salivary protein Bsp9 (whose expression is induced by TYLCV) that can effectively inhibit the plant defence response to whitefly infestation. Bsp9 interacts with a resistance-related transcription factor WRKY33. The plant immune regulation by Bsp9 affects whitefly fitness, thereby leading to a possible enhancement of virus transmission. Our research uncovered how begomoviruses manipulate whitefly effectors to promote virus transmission for worldwide invasion.

## 2. Material and methods

### (a) Plant and insect materials

Tomato (*Solanum lycopersicum*, Heinz 1706-BG, LA4345) seeds were ordered from Tomato Genetic Resource Center, University of California, Davis, USA, and propagated. Seeds of tomato and *Nicotiana benthamiana* were grown in a greenhouse at 25°C with a 12 L : 12 D cycle and young seedlings of three to four true leaf stages were used.

*Arabidopsis thaliana* (ecotype Col-0) was used for *Agrobacterium*-mediated transformation; the *Arabidopsis* *wrky33* mutant was given by Prof. Jinlong Qiu (Institute of Microbiology, Chinese Academy of Sciences)*.* The plant binary vectors *35S:GUS*, *35S:YFP*, *35S:Bsp9-YFP*, *35S:Bsp9-HA* were constructed based on PCR. Plasmids were introduced into *Agrobacterium tumefaciens* strain EHA105 by electrotransformation and *Arabidopsis* transformations were performed according to the floral-dipping method [26].

Whiteflies (*B. tabaci* MEAM1/B) were maintained on tomatoes in a growth chamber at 25°C with a 14 L : 10 D cycle and 65% relative humidity.

### (b) Transcriptome sequencing and data analysis

Total RNA was isolated from viruliferous or virus-free whitefly samples using TRIzol reagent (Invitrogen) according to the manufacturer's instructions and used for library construction and sequencing on Illumina HiSeq X Ten platform at the Annoroad Gene Technology Company (Beijing, China). The de novo assembly of RNA-seq was conducted using the Trinity platform. Illumina sequence data were selectively filtered using SolexaQA to remove read lengths less than 35 bp and low-quality sequence at each nucleotide. Clean reads of Illumina sequence data were mapped by Bowtie2. Raw counts for each predicted gene were calculated as reads per kilobase of exon model per million mapped fragments (FPKM). Based on these statistical analyses, genes with *p* < 0.01 and log2 (fold change) value of RPKM greater than 1 were considered to be significant differentially expressed genes (DEGs). DEGs between viruliferous and virus-free whiteflies were identified and mapped to whitefly salivary gland transcriptome using tBLASTn. Finally, we got unigenes upregulated by TYLCV in salivary glands. Trinotate and ORF (opening reading frame) Finder were used for performing the functional annotation of unigenes and ORFs. We narrowed the range of the genes by the length of ORFs between 200 and 600 bp. SignalP 4.0 program was used to predict the presence of signal peptides and cleavage sites in the amino acid sequences encoded by the ORFs found in salivary gland ESTs. Subsequently, proteins with a signal peptide were predicted to contain at least one transmembrane domain by TMHMM Server2.0 and therefore more likely to remain in the membrane of the salivary gland cell during secretion. Besides these, a protein without or with one transmembrane domain included in the part of predicted signal peptide would be considered as a secreted protein, as well as a potential salivary protein.

### (c) Virus inoculation

Tomato yellow leaf curl virus (TYLCV-SH2; GenBank accession no. AM282874) was kindly provided by Prof. Xue-Ping Zhou (Institute of Plant Protection, Chinese Academy of Agriculture Sciences, China). Tomato seedlings were inoculated with TYLCV by agro-inoculation [27]. Virus-inoculated tomatoes were cultivated in the growth chamber at 25°C.

### (d) Viral DNA measurement

Total genomic DNA was extracted from systemically infected leaves, and viral DNA was detected by real-time PCR with TYLCV-specific primers as well as the *Arabidopsis* *α-tubulin2* (At5g62690)-specific primers or tomato tubulin-specific primers as endogenous controls. Three biological replicates were used in this experiment.

### (e) Whitefly bioassay

The two-choice experiments with MEAM1 (Middle East-Asia Minor 1) *B. tabaci* were performed as described previously [9]. The preference of *B. tabaci* was compared in bioassays between two *Arabidopsis* genotype lines. Two six-week-old plants of similar size and leaf numbers were placed in a cage (40 × 40 × 40 cm) 20 cm apart. Two hundred adult whiteflies were captured and placed on ice for 1–2 min to temporarily stun them. They were then released from a point equidistant from the two plants. After 20 min whitefly free-choice, the number of whiteflies on each of the two plants was recorded. For one genotype, eight plants were used in each bioassay with three replicates.

The MEMA1 whitefly oviposition experiment was performed as described in Li *et al.* [9]. Each cohort of three male and female adult whiteflies was released into a leaf-clip cage that enclosed a single leaf of a six-week-old plant. Whitefly eggs on each leaf were counted after infestation for 10 days using a microscope. Eight plants of each line were used in the experiment. The MEMA1 whitefly nymph development experiment was then performed as described in Li *et al.* [9]. Each of 16 female adults were released into a leaf-clip cage that enclosed a single leaf of a six-week-old plant. The number of nymphs on the *Arabidopsis* leaves was counted after 20 days using a microscope. Eight biological replicates were conducted in this experiment.

### (f) Whitefly infestation

To investigate the effect of whitefly infestation on virus accumulation, three-week-old tomato plants were first infected by TYLCV for 14 days with the same virus load, then infested by MEMA1 whiteflies. After 3 days of whitefly infestation, all whiteflies were gently removed from the plants. TYLCV-infected tomato plants without whitefly infestation were used as control check (CK). Tomato plant samples were taken after one week of whitefly infestation.

For gene expression in whitefly-infested plants, leaves of healthy *Arabidopsis* plants were placed inside leaf-clip cages. Fifty adult whiteflies were captured and released into each cupped leaf. Leaf samples were collected after whitefly feeding at the indicated time points.

For the detection of Bsp9 secretion from whitefly to tomato, 500 adult whiteflies were released into a leaf-clip cage that enclosed a single leaf of a four-week-old plant and infested for 72 h before plant sampling.

For the detection of virus transmission by whitefly, virus-free adult whiteflies were placed on TYLCV-infected tomato plants for 48 h of virus acquisition. Thirty viruliferous whiteflies were then captured and released onto a three-week-old *Arabidopsis* plant grown on Murashige and Skoog medium and enclosed in a leaf-clip cage. After 72 h of whitefly-to-plant virus transmission, whiteflies were then gently removed and plant samples were collected to isolate individual DNA.

### (g) Quantitative RT-PCR

Total DNA of tomato leaves was isolated by the CTAB method (cetyltrimethyl ammonium bromide). Total RNA of whitefly was extracted using TRIzol reagent (Invitrogen, Carlsbad, CA, USA) according to the manufacturer's instructions. Total RNA of tomato leaves was isolated by plant RNA purification reagent (Invitrogen, Carlsbad, CA, USA) [28]. Total RNA of 800 ng for each sample was used for reverse transcription with TransScript One-Step gDNA Removal and followed by cDNA synthesis (Synthesis SuperMix, Transgen, China). Quantitative PCR was performed on a Bio-Rad CFX96 real-time PCR system with Thunderbird™ SYBR qPCR mix (TOYOBO). Four independent biological samples were analysed for each experiment and three independent experiments were performed and similar results were observed. The primers used for mRNA detection of target genes by real-time PCR are listed in electronic supplementary material, table S1.

### (h) Luciferase activity assay

*Arabidopsis* *PDF1.2 promoter*: *luciferase* was used as a reporter construct. Candidate whitefly salivary protein genes were driven by *Cauliflower mosaic virus* (CaMV) *35S promoter*: genes as effector constructs. Leaves of *N. benthamiana* were agroinfiltrated with the indicated agrobacterium stains which contain individual constructs. Infiltrated leaves were harvested after 2 days' treatment and the luciferase activity was quantified by a microplate reader.

Synthetic Pep1 (1 µM) peptide was used as an elicitor to activate plant immunity for 3 h before sampling. Each treatment was repeated eight times in one experiment. The experiment was repeated twice with similar results. The fold of luciferase activation by Pep1 was calculated against control group without Pep1 treatment. *35S:YFP* was used as the vector control.

For luciferase complementation-based protein interaction assay, *Agrobacterium* carrying the indicated constructs were infiltrated into *N. benthamiana* leaves and the luciferase imaging assays were performed 48 h after infiltration [29].

### (i) Yeast two-hybrid analysis

For this, the Arabidopsis Mate and Plate Library was used (Clontech). Full-length protein of Bsp9 was cloned into the pGBT9 vector to generate BD-Bsp9 as a bait vector and putative interaction was screened by following the manufacturer's protocol (Matchmaker Gold Yeast Two-Hybrid System, Clontech). To further confirm the interaction between Bsp9 and WRKY33, the yeast strain Y2H Gold was co-transformed with BD-Bsp9 and AD-WRKY33 constructs and plated on SD-Leu-Trp selective dropout medium. Colonies were transferred onto SD-Leu-Trp-His plates to verify positive clones. The binding domain vector (BD) pGBKT7 and activation domain vector (AD) pGADT7 were used as negative controls.

### (j) Bimolecular fluorescence complementation assay

All constructs were transferred into *Agrobacterium* C58C1 competent cells. The bimolecular fluorescence complementation (BiFC) assay was performed, as previously described in Sun *et al.* [30]. Agrobacterial cells containing indicated constructs were infiltrated into three-week-old *N. benthamiana* leaves. Fluorescence was observed owing to the complementation of Bsp9 fused with the cEYFP and WRKY33 fused with nEYFP. Images of fluorescence were taken by confocal microscopy (Leica SP8) after 48 h incubation. Plant nuclei were stained with DAPI (4′,6-diamidino-2-phenylindole) infiltrated into leaves 30 min prior to detection.

For competitive inhibition assay, *A. tumefaciens* strains containing expression vectors for cEYFP-MPK6 + nEYFP-WRKY33 and *35S:Bsp9* were co-injected into *N. benthamiana* leaf cells and kept in the dark for 2 days. The control was co-injected with the same volume MMA buffer as *35S:Bsp9*. Co-expression with the same volume *35S:GUS* was used as a negative control. Fluorescence intensity was measured by ImageJ.

### (k) Antibody preparation

The DNA fragment of Bsp9 was cloned into pET-28a (+) vector to generate 6×His-Bsp9 fusion construct. His-Bsp9 protein was purified using Ni-nitrilotriacetate (Ni-NTA) agarose (Qiagen) according to the manufacturer's instructions. Then, His-Bsp9 protein was injected into rabbit and the corresponding polyclonal antibody was generated by the Animal Center of Institute of Genetics and Developmental Biology, Chinese Academy of Sciences.

### (l) Protein extraction and Western blot

Protein was extracted from plants with extraction buffer (20 mM Tris–HCl pH 8.0, 100 mM NaCl, 10 mM MgCl_2_, 50 mM DTT, 0.5 mM PMSF and protease inhibitor cocktail). Equal amounts of total protein were separated on 10% SDS–polyacrylamide gels and transferred to a polyvinylidene difluoride membrane (Millipore) [30]. Bsp9 protein was detected by Western blot analysis with anti-Bsp9 polyclonal antibody.

### (m) Data analysis

Differences in TYLCV accumulation levels, gene expression levels, whitefly performance and relative fluorescence intensity and relative luciferase activity were determined using Student's *t*-tests for comparing two treatments or two lines. Differences in whitefly choice between different lines were analysed by nonparametric Wilcoxon-matched pair tests (with two dependent samples). All tests were carried out with Excel and GraphPad Prism.

### (n) Accession numbers

Sequence data from this work can be found in Genebank/EMBL or the *Arabidopsis* Information Resource (www.Arabidopsis.org) under the following accession numbers: TYLCV-SH2 (AM282874), AtPDF1.2 (AT5G44420), AtPEPR1 (AT1G73080), AtWRKY33 (AT2G38470), AtTPS10 (At2G24210), AtTPS14 (AT1G61680), AtTPS18 (AT3G14520), AtTPS20 (AT5G48110), MED whitefly Bsp9 (MH744980) and MEAM1 whitefly Bsp9 (MH744981).

## 3. Results

### (a) Whitefly infestation increases the accumulation of tomato yellow leaf curl virus

**Figure 1.**
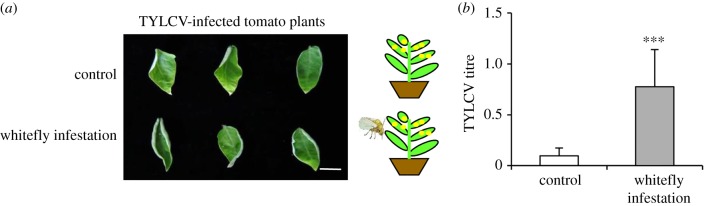
Whitefly infestation increases pathogenesis and accumulation of TYLCV. (*a*) TYLCV-infected tomato plants infested with whiteflies for one week or without (as control). Scale bars = 2 cm. (*b*) Relative TYLCV titre in the infected tomato leaves. Error bars represent +s.d. (*n* = 4). Asterisks indicate significant differences (***p < 0.001, Student's *t*-test).

We inoculated tomato with TYLCV, a begomovirus transmitted by an invasive MEAM1 whitefly, and examined the symptoms of infected plants. TYLCV-infected tomatoes showed mild yellowish symptoms at 14 days after inoculation while using the agroinfiltration method alone. Interestingly, TYLCV-infected tomato plants, followed by infestation of MEMA1 whiteflies for 3 days, exhibited more obvious yellowish and upward curling leaf symptoms at 7 days after infestation of whitefly compared with mock control (figure 1*a*). To see whether accumulation levels of TYLCV in virus-infected plants are increased due to whitefly infestation, we further performed quantitative PCR analysis for TYLCV titre. The relative level of TYLCV was obviously 10-fold higher in whitefly-infested plants (figure 1*b*). Therefore, the infestation of whitefly enhances the pathogenesis of its transmitted begomovirus on tomato.

### (b) Comparative transcriptomic screening of whitefly salivary effectors

One hypothesis for the enhancement of TYLCV pathogenesis by the infestation of whitefly is that TYLCV could induce accumulation of whitefly salivary effectors and, in turn, inhibit host defence against virus. To identify candidate whitefly salivary effectors induced by TYLCV, we firstly undertook a comparative transcriptomic analysis of viruliferous whitefly versus virus-free whitefly (electronic supplementary material, figure S1). High-quality total RNA samples of viruliferous whitefly and virus-free MEAM1 whitefly were extracted, and de novo sequencing was conducted with the Illumina sequencing platform. Through filtering the adaptors and low-quality sequences, assembling by Trinity and mapping by Bowtie2, we finally got 86 428 unigenes based on the raw data of scaffolds. Among them, expression levels of 53 353 unigenes were induced by TYLCV at least twofold. Comparing with the whitefly salivary gland dataset, only 1.4% of TYLCV-induced unigenes (778/53 353) were found in salivary glands [31]. Taking into consideration that most effectors are putative secreted proteins which possess a suitable opening reading frame size, an N-terminal signal peptide and have no transmembrane regions, finally, we cloned 10 full-length cDNA of TYLCV-induced candidate salivary effector proteins for further functional characterizations (electronic supplementary material, table S2).

### (c) Whitefly salivary protein Bsp9 suppresses plant immune response

**Figure 2.**
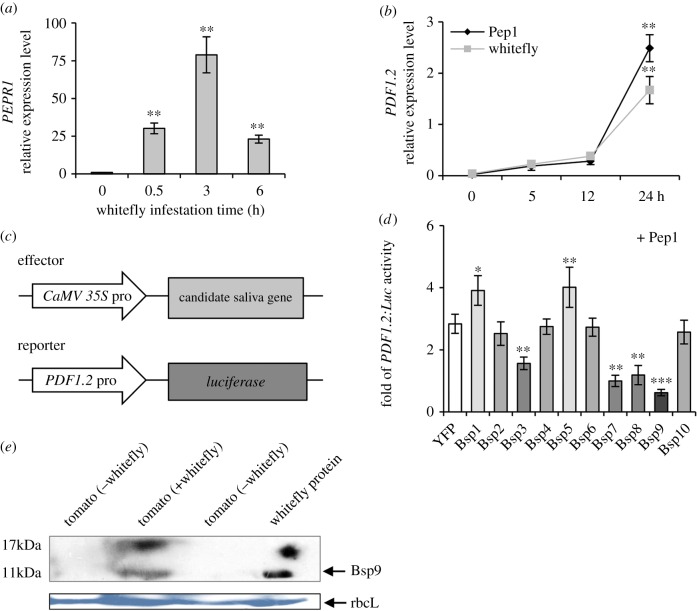
Functional characterization of TYLCV-induced whitefly salivary effectors. (*a*) Relative expression level of *PEPR1* that encoded a receptor to recognize DAMPs upon infestation of whitefly in *Arabidopsis*. Three-week-old Col-0 plants were infested with whiteflies for the indicated time. Values are mean ± s.d. (*n* = 4). (*b*) Relative induction of anti-herbivory marker gene *PDF1.2* upon infestation of whitefly or Pep1 treatment. Three-week-old Col-0 plants were infested with whiteflies or 1 µM DAMP molecular peptide Pep1 for the indicated time. Values are mean ± s.e. (*n* = 4). (*c*) Schematic diagram of whitefly salivary effectors’ functional screening system showing the effector and luciferase reporter constructs used in (*d*). The *Arabidopsis* *PDF1.2* promoter-driven *luciferase* was used as a reporter. *CaMV 35S* promoter-driven whitefly salivary protein genes were used as effector constructs. (*d*) Whitefly (*Bemisia tabaci*) salivary proteins (Bsp1–Bsp10) were screened for their ability to affect DAMP-induced plant immunity on *Nicotiana benthamiana* leaf. Immunity activator Pep1 (1 µM) was spread for 3 h before sampling. The fold increase of luciferase activation by Pep1 was calculated against a control group without Pep1 treatment. *35S:YFP* was used as a control. Asterisks indicate significant differences in fold increase of luciferase activation between control and candidate whitefly saliva protein genes. Bars represent means ± s.e (*n* = 8) (*p < 0.05; **p < 0.01, ***p < 0.001; Student's *t*-test for all the experiments). (*e*) Detection of Bsp9 protein in plants. Tomato plants were infested with whitefly for 72 h and all whiteflies in leaves were removed before sampling. Bsp9 protein was detected by Western blot using polyclonal antibody anti-Bsp9. Tomato leaves without whitefly infestation were used as a negative control, and whitefly total protein was used as a positive control. Stained gel bands of the large subunit of Rubisco (rbcL) were used as a loading control. (Online version in colour.)

As a functional analysis platform for whitefly salivary proteins has rarely been reported, we sought to establish a suitable screening and reporter system to identify whitefly effectors. We observed that the expression of a PTI membrane receptor *PEPR1* was rapidly induced upon whitefly infestation in *Arabidopsis* (figure 2*a*). Furthermore, another downstream defensive marker gene *Arabidopsis* *PDF1.2* was also highly induced upon whitefly infestation or the treatment with Pep1 polypeptide, a ligand for PEPR1/2 receptors (figure 2*b*; electronic supplementary material, figure S3). These results indicated that Pep1 treatment can mimic the stimulation of whitefly infestation on plants and the expression level of *PDF1.2* may represent the level of plant immune response to whitefly. Accordingly, we developed a novel system consisting of an effector and a reporter system together to functionally identify a whitefly salivary immunity regulator (as shown in figure 2*c*). Each of these two plasmids was transformed into *A. tumefaciens* and co-inoculated leaves of *N. benthamiana* by agroinfiltration. By measuring the activity of the *PDF1.2* promoter upon co-expression of tested protein, it is convenient for high-throughput screening of whitefly salivary effectors which can repress plant immune response induced by Pep1. As expected, we found that Pep1 treatment stimulates activity of the *PDF1.2* promoter (figure 2*d*). Interestingly, four salivary effectors repressed the Pep1-triggered activation of *PDF1.2* promoter. By contrast, two salivary effectors improved this activity. The preliminary functions of all tested salivary proteins are listed in electronic supplementary material, table S2. Among those, the degree of immune repression by Bsp9 was the highest (figure 2*d*). Thus, we chose Bsp9 for a detailed downstream analysis on the mechanism of its suppression of PTI.

### (d) Bsp9 secretes from whitefly into plant cells

*Bsp9* encodes a small protein with 112 amino acid residues and a molecular weight of 12.4 kDa (electronic supplementary material, table S3). The N-terminal of Bsp9 protein contains a 25 amino acid residue signal peptide with a transmembrane domain (electronic supplementary material, figure S4*a*,*b*). The secondary structure of Bsp9 mature protein is rich in α-helix. To confirm whether Bsp9 protein can be secreted from whitefly into plant cells, we detected Bsp9 protein in whitefly-infested tomato by immunoblot using anti-Bsp9 polyclonal antibody. The Bsp9 protein was indeed detected in tomato leaves infested by whitefly, as well as protein extracted from adult whitefly (figure 2*e*). As expected, there is no detectable signal in tomato leaves without whitefly feeding. To further check the subcellular localization of Bsp9 in plants, Bsp9-YFP fusion protein was expressed and highly accumulated in the cytoplasm of *N. benthamiana* leaf cells (electronic supplementary material, figure S4*c*).

### (e) Whitefly salivary protein Bsp9 interacts with *Arabidopsis* transcription factor WRKY33

**Figure 3.**
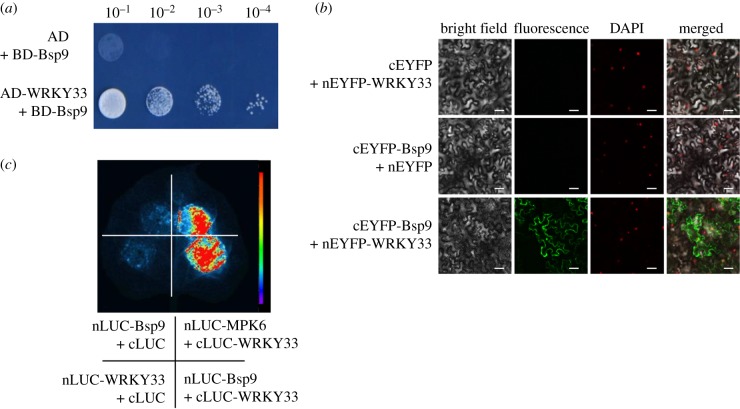
Interaction between whitefly effector protein Bsp9 and WRKY33. (*a*) Interaction between Bsp9 and WRKY33 in the yeast two-hybrid assay. Yeast strain Y2H Gold co-transformed with the indicated plasmids was spotted on synthetic medium SD-Leu-Trp-His. The empty activation domain vector (AD) pGADT7 was used as a negative control. (*b*) BiFC assay of the interaction between Bsp9 and *Arabidopsis* WRKY33. Nuclei of tobacco leaf epidermal cells were stained by DAPI. Unfused cEYFP or nEYFP was used as a negative control. cEYFP, C-terminus of YFP; nEYFP, N-terminus of YFP. Scale bars = 50 µm. (*c*) WRKY33 interacts with Bsp9 *in vivo* in the luciferase complementation assay. The *Agrobacterium* carrying the indicated constructs were infiltrated into *N. benthamiana* leaves and the luciferase imaging was taken 48 h after infiltration.

To investigate the molecular mechanism of how whitefly salivary protein Bsp9 promotes TYLCV accumulation in plants, we sought to identify Bsp9-targeted host factor(s). Therefore, a yeast two-hybrid experiment was conducted to screen an *Arabidopsis* cDNA library by using Bsp9 as the bait, and an immunity-related transcription factor AtWRKY33 was found as a putative positive interactor. Yeast transformants carrying AD-WRKY33 and BD-Bsp9 were able to grow on SD-Leu-Trp-His selection plates with 2 mM 3-amino-1,2,4-triazole, whereas yeast transformants carrying AD and BD-Bsp9 constructs were unable to do so (figure 3*a*). To confirm the interaction between WRKY33 and Bsp9 proteins *in vivo*, we performed a BiFC assay in *N. benthamiana*. The N-terminus of the yellow fluorescent protein was fused in-frame to WRKY33 (nEYFP-WRKY33) and C-terminus YFP was fused to Bsp9 (cEYFP-Bsp9). The constructs were transiently expressed in *N. benthamiana* leaf cells by *Agrobacterium* co-infiltration. A direct interaction between WRKY33 and Bsp9 was observed in the form of cytoplasmic speckles, which altered the nucleus localization of WRKY33 as a functional transcription factor to regulate downstream defensive gene expression (figure 3*b*). No fluorescence was detected when cEYFP-Bsp9 or nEYFP-WRKY33 was co-expressed with nEYFP or cEYFP as a negative control. A split-luciferase complementation assay was further performed to confirm the interaction *in vivo*. *Agrobacterium* carrying the constructs of nLUC-Bsp9 and cLUC-WRKY33 were infiltrated into *N. benthamiana* leaves, and the intensity of luminescence was increased only by the combination of nLUC-Bsp9 and cLUC-WRKY33 compared with the vector control (figure 3*c*). Taken together, these results consistently prove that Bsp9 interacts with WRKY33.

### (f) Bsp9 may disrupt the interaction between MPK6 and WRKY33

WRKY33 is an essential transcription factor in response to the attack of pathogens, but how it regulates immunity against whitefly and begomovirus is unknown. Bsp9 interacts with WRKY33 in the cytoplasmic bodies, raising a possibility that Bsp9 competes with MPK3 or MPK6 for the interaction with WRKY33.

**Figure 4.**
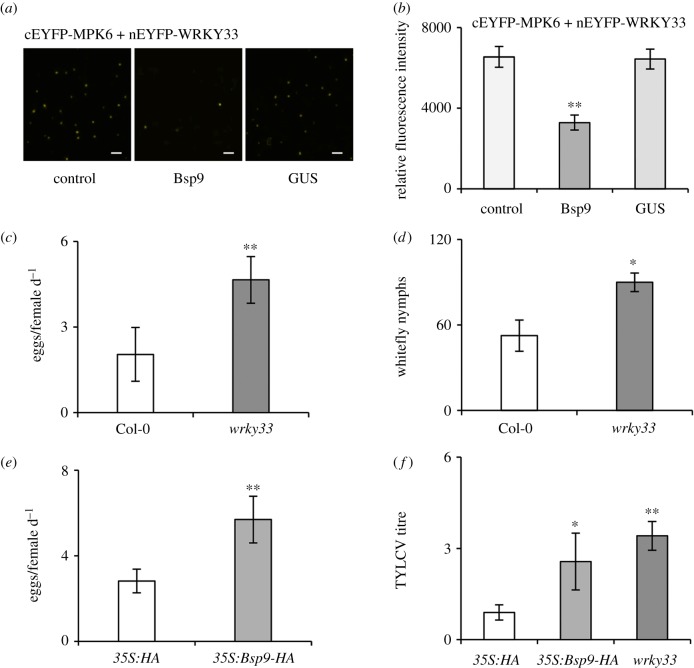
Bsp9 inhibits WRKY33-mediated anti-whitefly resistance and increases whitefly performance and TYLCV transmission in *Arabidopsis*. (*a*) Bsp9 interferes with the interaction between MPK6 and WRKY33 in the modified BiFC assay. *Agrobacterium tumefaciens* strains containing expression vectors for fusion proteins of MPK6 and WRKY33, together with *35S:Bsp9*, were co-injected into *N. benthamiana* leaf cells and kept for 2 days before observation. Yellow fluorescence was observed owing to the interaction between cEYFP-MPK6 and nEYFP-WRKY33. The control was treated with a similar volume of infiltration buffer. Co-expression with a *35S*:*β-glucuronidase* (*35S:GUS*) was used as a negative control. (*b*) Quantitative data of EYFP fluorescence intensity show effects of Bsp9 on the interaction of WRKY33 with MPK6. Fluorescence intensity was measured by ImageJ. Bars represent means ± s.e. (*n* = 12). (*c*) Daily number of eggs laid per female whitefly on Col-0 and *wrky33* plants. Values are means ± s.d. (*n* = 8). (*d*) Number of late fourth whitefly nymphs on Col-0 and *wrky33* plants. Values are means ± s.d. (*n* = 8) (*p < 0.05; **p < 0.01; Student's *t*-tests for all the experiments except the whitefly choice experiments, which were analysed by the Wilcoxon-matched pairs test). (*e*) Daily number of eggs laid per female whitefly on *35S:HA* and *35S:Bsp9-HA* plants. Values are means ± s.d. (*n* = 8). (*f*) Relative virus amount of whitefly transmission into *Arabidopsis* plants. Viruliferous whiteflies were allowed to feed on *Arabidopsis* for 72 h. *35S:HA* transgenic *Arabidopsis* plants infested with non-viruliferous whitefly were used as control. TYLCV titre was quantified by qPCR. Values are means ± s.d. (*n* = 4) (**p < 0.01; Student's *t*-test for all the experiments except the whitefly choice experiments, which were analysed by the Wilcoxon matched pairs test).

A modified BiFC competitive protein-binding assay was used to test this hypothesis. *Agrobacterium tumefaciens* strains containing expression vectors for fusion proteins of MPK6 and WRKY33, together with *35S:Bsp9*, were co-injected into *N. benthamiana* leaf cells. Yellow fluorescence was observed owing to the interaction between cEYFP-MPK6 and nEYFP-WRKY33. Co-expression with a *35S:β-glucuronidase* (*35S:GUS*) was used as a negative control. The interaction strength of MPK6-WRKY33 as indicated by EYFP fluorescence intensity was significantly decreased after the addition of Bsp9 protein (figure 4*a*,*b*). A negative control of GUS co-expression did not affect the interaction between MPK6 and WRKY33. These results demonstrate that Bsp9 interferes with the interaction between MPK6 and WRKY33, and, therefore, might disrupt a signal transduction event.

### (g) WRKY33 is essential for *Arabidopsis* anti-whitefly resistance

Considering the reduced interaction between MPK6 and WRKY33 due to the interference of Bsp9, we hypothesized that Bsp9 may affect the WRKY33-mediated immune response. However, whether WRKY33 can mediate plant innate immunity against whitefly is still unclear. To test the regulation of WRKY33 in response to whitefly, we performed a whitefly two-choice preference assay. Electronic supplementary material, figure S5*a* shows that more whiteflies were attracted to *wrky33* plants compared with Col-0 plants. Our previous work has indicated that suppression of JA-regulated repellent terpene biosynthesis in *Arabidopsis* makes the host more attractive to the whitefly vector [9], and thus we examined the expressions of *Arabidopsis* *Terpene Synthase* (*TPS*) genes such as *TPS10*, *TPS14*, *TPS18* and *TPS20*. As expected, the expression levels of *TPS10*, *TPS18* and *TPS20* in the *wrky33* mutant were greatly reduced compared with those of Col-0 plants (electronic supplementary material, figure *S*5*b*). In addition to the increased whitefly attraction, more eggs were laid by whiteflies on *wrky33* mutants than on Col-0 plants (figure 4*c*). Furthermore, late fourth whitefly nymphs were much more prevalent on *wrky33* than on Col-0 plants (figure 4*d*). That whiteflies preferred and performed better on *wrky33* plants indicates that WRKY33 is essential for plant resistance against whitefly. Taken together, our results imply that Bsp9 inhibits WRKY33-mediated anti-whitefly resistance.

### (h) Bsp9 increases whitefly performance and transmission of TYLCV

Previous studies demonstrated that plants pre-infested with TYLCV-viruliferous whitefly could attract more vector insects [32]. Transient expression of Bsp9 inhibits WRKY33-mediated resistance, but whether the suppression of Bsp9 affects whitefly preference for better virus transmission is unknown. Thus, we generated transgenic plants overexpressing *Bsp9.* Intriguingly, we found that *35S:Bsp9-HA* stable transgenic lines were more attractive to whiteflies compared with *35S:HA* vector control plants (electronic supplementary material, figure S5*c*). Besides increased whitefly attraction, daily number of eggs laid per female whitefly on *35S:Bsp9-HA* plants showed a significant increase compared with that of vector control plants, indicating that Bsp9 suppresses plant resistance against whitefly (figure 4*e*). The promoted whitefly preference and performance raised the conjecture of whether the manipulation of whitefly behaviours by Bsp9 can eventually affect the viral transmission efficiency from whitefly to plants. To test this, we detected the amount of whitefly virus transmission into *Arabidopsis* plants which were fed on by TYLCV-viruliferous whiteflies for 72 h, at which time point no viral transcription could be detected in plants (electronic supplementary material, figure S6). Results showed that TYLCV titre in *35S:Bsp9*-*HA* plants was threefold higher than that in vector control plants (figure 4*f*). Consistent with the promoted whitefly performance on the *wrky33* mutant, the amount of virus transmission to *wrky33* plants increased fourfold compared to that of vector control plants. Based on the above results, we conclude that the whitefly salivary protein Bsp9 suppresses WRKY33-mediated immunity to increase whitefly preference, performance and, in turn, eventually increases virus transmission.

## 4. Discussion

How have whiteflies become a successful supervector, able to transmit 300 species of viruses? [1] In this report, we provide a new layer of insight, in which a virus-induced whitefly salivary effector Bsp9 benefits both vector and virus. We have provided several lines of evidence that Bsp9 is a critical salivary effector in enhancing virus transmission. First, Bsp9 can effectively suppress the plant immune response activated by whitefly infestation (figure 2). Second, Bsp9 can be secreted into plants and interacts with the transcription factor WRKY33 (figures 2*e* and 3; electronic supplementary material, figure S4). Third, Bsp9 alters the localization of WRKY33 and affects the interaction between WRKY33 and MPK6, interfering with the WRKY33-mediated plant innate immunity against pathogen and insects (figures 3*b* and 4). Fourth, Bsp9 can effectively increase whitefly preference, performance and also TYLCV transmission (figure 4; electronic supplementary material, figure S5). Fifth, genomic analysis shows that Bsp9 is highly conserved in two invasive species of whitefly, MEAM1 and MED (electronic supplementary material, table S3). The coding region of the Bsp9 gene is highly conserved in these two whitefly species and there is only one non-synonymous mutation, indicating the critical role of Bsp9 for whitefly fitness on host plant adaption. Meanwhile, the high mutation rates in intron and untranslational regions suggest that a possible transcriptional level regulation may play an important role for whiteflies in response to biotic stresses, e.g. TYLCV infection in this report. Nevertheless, it is essential to clone Bsp9 homologues from native whiteflies and other Aleyrodoidea for understanding its role in whitefly invasion and begomoviral diseases pandemic in the world. With the rapid development of CRISPR/CAS-based single-base gene-editing technology [33], it is very interesting to look at the significance of single nucleotide differences of *Bsp9* for the tripartite interactions of whitefly–plant–virus and environmental stress responses between MEAM1 and MED whiteflies.

While microbial pathogen effectors have been extensively studied for a long time, a number of functional approaches to identify effectors secreted by insects have only recently attracted attention [3–5]. However, the majority of the salivary gland transcripts encode small proteins, which lack sequence similarity to function-annotated proteins. Transcriptomic and proteomic analyses of aphid salivary glands or aphid saliva, combined in some cases with RNAi and plant stable overexpression approaches, have revealed the presence of potential effector proteins, such as Mp1 (PIntO1) and Mp2 (PIntO1), Mp10, Mp42, Mp55, Mp56, Mp57 and Mp58 in aphids [3,34–36]. However, most of the functional studies of insect effectors are designed on the basis of plant–pathogen interaction systems. For example, in most of these analyses, host immune responses are triggered by applying a pathogen-derived elicitor such as flg22 or elf18 [17], like aphid effector Mp10, which was functionally identified by the suppression of the oxidative burst induced by the bacterial elicitor flg22 [4]. But, this does not reflect the nature of plant–insect interaction. In the interaction between insects and plants upon feeding damage, DAMPs (damage-associated molecular patterns) are more likely to reflect the real situation of plant–insect interaction. In our study, we found that a DAMP Pep1 treatment could mimic the whitefly infestation on a plant, and the expression level of *PDF1.2* may reflect changes of plant defence specific to whitefly. We developed a novel screening platform and successfully identified four candidate effectors, from the invasive MEAM1 whitefly, which can suppress Pep1-induced expression of *PDF1.2*. Among them, Bsp9, which has the strongest suppression efficacy of plant immune responses, has been demonstrated as a functionally characterized whitefly salivary protein able to disrupt WRKY33-mediated immune signalling. Nevertheless, in the future, it will be promising to check *Bsp9* knockout/down whitefly to look for other roles in whitefly biology and whitefly–plant interaction. Also, it will be interesting to check the effect of *wrky33* mutation on the *Bsp9* overexpression lines to further confirm whether WRKY33 is the major target of Bsp9.

Previously published studies show that WRKY33 is a pathogen-inducible transcription factor, whose expression is regulated by the MPK3/MPK6 cascade. In *Arabidopsis*, WRKY33 functions in response to PTI signalling and also reprogramming of the expression of camalexin biosynthetic genes. *WRKY33* expression is regulated by the MAPK cascades [22]. Previous studies found that MAPKs can be activated by whitefly-mediated inoculation of TYLCV [37,38]. MPK3/MPK6 can phosphorylate WRKY33, the latter then binds to not only its own promoter but also the *PAD3* promoter and positively regulates the expression of its bound genes. WRKY33 binds camalexin biosynthetic genes which encode proteins to produce defensive metabolites for pathogen resistance in *Arabidopsis* [39,40]. WRKY33 drives the metabolic flow to camalexin production challenged by pathogens. The interaction between WRKY33 and MPK3/6 in the nucleus cells plays an important role in the MAPK–WRKY33 cascade. WRKY33 is subject to post-translational modification by MAPK4 that is involved in salicylic acid-mediated responses as well [41,42]. In this study, WRKY33 is identified as regulating plant immune response against whitefly in *Arabidopsis*. Interestingly, we also show that WRKY33 is involved in the regulation of biosynthesis of terpenes, the major chemical communication signals between plant and whitefly. Both *Arabidopsis* *TPS10* and *TPS14* encode synthases of β-myrcene, which is an efficient repellent against MEAM1 whitefly. The deficiency of *Arabidopsis* *TPS10* due to mutation of *WRKY33* is more attractive to whitefly, coincidentally with the reduced *TPS10* expression in another transcriptional regulator *MYC2* mutant *Arabidopsis* [9]. WRKY33 controls toxic camalexin production through its interaction with MPK3/6, as well as another MAPK, MPK4, which in turn activates the expression of *PAD3* [43,44]. Thus, WRKY33 may participate in the biosynthesis regulation of the phytoalexin camalexin to mediate resistance to both pathogens and whiteflies.

## 5. Conclusion

In summary, we identify a new strategy for vector-borne viruses to conquer host immunity. In a healthy plant, whitefly feeding activates the PTI which triggers the activation of MAPKs and the expression of plant defensive genes. In begomovirus-infected whiteflies, whitefly saliva proteins can be induced by TYLCV and are secreted into plants during feeding. One of whitefly effectors Bsp9 can effectively suppress the expression of marker gene *PDF1.2*. Bsp9 interacts with plant transcription factor WRKY33 and interferes with the interaction of WRKY33 and MPK6, and therefore, suppresses the WRKY33-induced immune response. Thus, begomovirus might manipulate saliva effectors to suppress host immune responses to benefit whitefly fitness and virus spread. However, the detailed mechanism of how TYLCV promotes the transcription of whitefly *Bsp9* is still a subject of study in the laboratory.

## Supplementary Material

Supplementary information

## Data accessibility

The datasets supporting this article have been uploaded as part of the electronic supplementary material.

## References

[1] Gilbertson RL, Batuman O, Webster CG, Adkins S 2015 Role of the insect supervectors *Bemisia tabaci* and *Frankliniella occidentalis* in the emergence and global spread of plant viruses. Annu. Rev. Virol. 2, 67–93. (10.1146/annurev-virology-031413-085410) 26958907

[2] Le Fevre R, Evangelisti E, Rey T, Schornack S 2015 Modulation of host cell biology by plant pathogenic microbes. Annu. Rev. Cell Dev. Biol. 31, 201–229. (10.1146/annurev-cellbio-102314-112502) 26436707

[3] Elzinga DA, Jander G 2013 The role of protein effectors in plant-aphid interactions. Curr. Opin. Plant Biol. 16, 451–456. (10.1016/j.pbi.2013.06.018) 23850072

[4] Bos JI, Prince D, Pitino M, Maffei ME, Win J, Hogenhout SA 2010 A functional genomics approach identifies candidate effectors from the aphid species *Myzus persicae* (green peach aphid). PLoS Genet. 6, e1001216 (10.1371/journal.pgen.1001216) 21124944 PMC2987835

[5] Pitino M, Hogenhout SA 2013 Aphid protein effectors promote aphid colonization in a plant species-specific manner. Mol. Plant Microbe Interact. 26, 130–139. (10.1094/MPMI-07-12-0172-FI) 23035913

[6] Wang XW, Li P, Liu SS 2017 Whitefly interactions with plants. Curr. Opin. Insect Sci. 19, 70–75. (10.1016/j.cois.2017.02.001) 28521945

[7] Lee HR, Lee S, Park S, Van Kleeff PJ, Schuurink R, Ryu CM 2018 Transient expression of whitefly effectors in *Nicotiana benthamiana* leaves activates systemic immunity against the leaf pathogen *Pseudomonas syringae* and soil-borne pathogen *Ralstonia solanacearum*. Front. Ecol. Environ. 6, 90 (10.3389/fevo.2018.00090)

[8] Zhou XP 2013 Advances in understanding begomovirus satellites. Annu. Rev. Phytopathol. 51, 357–381. (10.1146/annurev-phyto-082712-102234) 23915133

[9] Li R et al 2014 Virulence factors of geminivirus interact with MYC2 to subvert plant resistance and promote vector performance. Plant Cell 26, 4991–5008. (10.1105/tpc.114.133181) 25490915 PMC4311212

[10] Aljbory Z, Chen MS 2018 Indirect plant defense against insect herbivores: a review. Insect Sci. 25, 2–23. (10.1111/1744-7917.12436) 28035791

[11] Wu JQ, Baldwin IT 2010 New insights into plant responses to the attack from insect herbivores. Annu. Rev. Genet. 44, 1–24. (10.1146/annurev-genet-102209-163500) 20649414

[12] Diezel C, Dahl CCV, Gaquerel E, Baldwin IT 2009 Different lepidopteran elicitors account for cross-talk in herbivory-induced phytohormone signaling. Plant Physiol. 150, 1576–1586. (10.1104/pp.109.139550) 19458114 PMC2705021

[13] Zhang T, Luan JB, Qi JF, Huang CJ, Li M, Zhou XP, Liu SS 2012 Begomovirus-whitefly mutualism is achieved through repression of plant defences by a virus pathogenicity factor. Mol. Ecol. 21, 1294–1304. (10.1111/j.1365-294X.2012.05457.x) 22269032

[14] Howe GA, Jander G 2008 Plant immunity to insect herbivores. Annu. Rev. Plant Biol. 59, 41–66. (10.1146/annurev.arplant.59.032607.092825) 18031220

[15] Dangl JL, Horvath DM, Staskawicz BJ 2013 Pivoting the plant immune system from dissection to deployment. Science 341, 746–751. (10.1126/science.1236011) 23950531 PMC3869199

[16] Wu J, Baldwin IT 2009 Herbivory-induced signalling in plants: perception and action. Plant Cell Environ. 32, 1161–1174. (10.1111/j.1365-3040.2009.01943.x) 19183291

[17] Zipfel C 2014 Plant pattern-recognition receptors. Trends Immunol. 35, 345–351. (10.1016/j.it.2014.05.004) 24946686

[18] Klauser D, Desurmont GA, Glauser G, Vallat A, Flury P, Boller T, Turlings TC, Bartels S 2015 The *Arabidopsis* Pep-PEPR system is induced by herbivore feeding and contributes to JA-mediated plant defence against herbivory. J. Exp. Bot. 66, 5327–5336. (10.1093/jxb/erv250) 26034129 PMC4526914

[19] Acevedo FE, Rivera-Vega LJ, Chung SH, Ray S, Felton GW 2015 Cues from chewing insects—the intersection of DAMPs, HAMPs, MAMPs and effectors. Curr. Opin. Plant Biol. 26, 80–86. (10.1016/j.pbi.2015.05.029) 26123394

[20] Huffaker A, Pearce G, Ryan CA 2006 An endogenous peptide signal in *Arabidopsis* activates components of the innate immune response. Proc. Natl. Acad. Sci. USA. 103, 10 098–10 103. (10.1073/pnas.0603727103) 16785434 PMC1502512

[21] Bonaventure G, Vandoorn A, Baldwin IT 2011 Herbivore-associated elicitors: FAC signaling and metabolism. Trends Plant Sci. 16, 294–299. (10.1016/j.tplants.2011.01.006) 21354852

[22] Meng X, Zhang S 2013 MAPK cascades in plant disease resistance signaling. Annu. Rev. Phytopathol. 51, 245–266. (10.1146/annurev-phyto-082712-102314) 23663002

[23] Kempema LA, Cui X, Holzer FM, Walling LL 2007 *Arabidopsis* transcriptome changes in response to phloem-feeding silverleaf whitefly nymphs. Similarities and distinctions in responses to aphids. Plant Physiol. 143, 849–865. (10.1104/pp.106.090662) 17189325 PMC1803730

[24] Erb M, Meldau S, Howe GA 2012 Role of phytohormones in insect-specific plant reactions. Trends Plant Sci. 17, 250–259. (10.1016/j.tplants.2012.01.003) 22305233 PMC3346861

[25] Heinrich M, Baldwin IT, Wu J 2011 Two mitogen-activated protein kinase kinases, MKK1 and MEK2, are involved in wounding- and specialist lepidopteran herbivore *Manduca sexta*-induced responses in *Nicotiana attenuata*. J. Exp. Bot. 62, 4355–4365. (10.1093/jxb/err162) 21610019 PMC3153688

[26] Zhang X, Henriques R, Lin SS, Niu QW, Chua NH 2006 *Agrobacterium*-mediated transformation of *Arabidopsis thaliana* using the floral dip method. Nat. Protoc. 1, 641–646. (10.1038/nprot.2006.97) 17406292

[27] Xie Y, Jiang T, Zhou XP 2006 Agroinoculation shows *Tobacco leaf curl Yunnan virus* is a monopartite begomovirus. Eur. J. Plant Pathol. 115, 369–375. (10.1007/s10658-006-9021-8)

[28] Ye J, Yang J, Sun Y, Zhao P, Gao S, Jung C, Qu J, Fang R, Chua NH 2015 Geminivirus activates *ASYMMETRIC LEAVES 2* to accelerate cytoplasmic DCP2-mediated mRNA turnover and weakens RNA silencing in *Arabidopsis*. PLoS Pathog. 11, e1005196 (10.1371/journal.ppat.1005196) 26431425 PMC4592220

[29] Li L, Kim P, Yu L, Cai G, Chen S, Alfano JR, Zhou JM 2016 Activation-dependent destruction of a co-receptor by a *Pseudomonas syringae* effector dampens plant immunity. Cell Host Microbe 20, 504–514. (10.1016/j.chom.2016.09.007) 27736646

[30] Sun Y et al 2017 Manipulation of auxin response factor 19 affects seed size in the woody perennial *Jatropha curcas*. Sci. Rep. 7, 40844 (10.1038/srep40844) 28102350 PMC5244365

[31] Su YL, Li JM, Li M, Luan JB, Ye XD, Wang XW, Liu SS 2012 Transcriptomic analysis of the salivary glands of an invasive whitefly. PLoS One 7, e39303 (10.1371/journal.pone.0039303) 22745728 PMC3379992

[32] Liu B, Preisser EL, Chu D, Pan H, Xie W, Wang S, Wu Q, Zhou X, Zhang Y 2013 Multiple forms of vector manipulation by a plant-infecting virus: *Bemisia tabaci* and *tomato yellow leaf curl virus*. J. Virol. 87, 4929 (10.1128/JVI.03571-12) 23408638 PMC3624301

[33] Cong L et al. 2013 Multiplex genome engineering using CRISPR/Cas systems. Science 339, 819–823. (10.1126/science.1231143) 23287718 PMC3795411

[34] Mondal HA 2017 Shaping the understanding of saliva-derived effectors towards aphid colony proliferation in host plant. Plant Biol. 60, 103–115. (10.1007/s12374-016-0465-x)

[35] Dicke M 2016 Induced plant volatiles: plant body odours structuring ecological networks. New Phytol. 210, 10–12*.* (10.1111/nph.13896) 26919694

[36] Schmelz EA 2015 Impacts of insect oral secretions on defoliation-induced plant defense. Curr. Opin. Insect Sci. 9, 7–15. (10.1016/j.cois.2015.04.002) 32846712

[37] Luan JB, Li JM, Varela N, Wang YL, Li FF, Bao YY, Zhang CX, Liu SS, Wang XW 2011 Global analysis of the transcriptional response of whitefly to *tomato yellow leaf curl China virus* reveals the relationship of coevolved adaptations. J. Virol. 85, 3330–3340. (10.1128/JVI.02507-10) 21270146 PMC3067855

[38] Gorovits R, Akad F, Beery H, Vidavsky F, Mahadav A, Czosnek H 2007 Expression of stress-response proteins upon whitefly-mediated inoculation of *Tomato yellow leaf curl virus* in susceptible and resistant tomato plants. Mol. Plant Microbe Interact. 20, 1376–1383. (10.1094/MPMI-20-11-1376) 17977149

[39] Birkenbihl RP, Diezel C, Somssich IE 2012 *Arabidopsis* WRKY33 is a key transcriptional regulator of hormonal and metabolic responses toward *Botrytis cinerea* infection. Plant Physiol. 159, 266–285. (10.1104/pp.111.192641) 22392279 PMC3375964

[40] Mao G, Meng X, Liu Y, Zheng Z, Chen Z, Zhang S 2011 Phosphorylation of a WRKY transcription factor by two pathogen-responsive MAPKs drives phytoalexin biosynthesis in *Arabidopsis*. Plant Cell 23, 1639–1653. (10.1105/tpc.111.084996) 21498677 PMC3101563

[41] Qiu JL et al. 2008 *Arabidopsis* mitogen-activated protein kinase kinases MKK1 and MKK2 have overlapping functions in defense signaling mediated by MEKK1, MPK4, and MKS1. Plant Physiol. 148, 212–222. (10.1104/pp.108.120006) 18599650 PMC2528087

[42] Gao M, Liu J, Bi D, Zhang Z, Cheng F, Chen S, Zhang Y 2008 MEKK1, MKK1/MKK2 and MPK4 function together in a mitogen-activated protein kinase cascade to regulate innate immunity in plants. Cell Res. 18, 1190–1198. (10.1038/cr.2008.300) 18982020

[43] Rodriguez MCS, Petersen M, Mundy J 2010 Mitogen-activated protein kinase signaling in plants. Annu. Rev. Plant Biol. 61, 621–649. (10.1146/annurev-arplant-042809-112252) 20441529

[44] Li B et al. 2015 Phosphorylation of trihelix transcriptional repressor ASR3 by MAP KINASE4 negatively regulates *Arabidopsis* immunity. Plant Cell 27, 839–856. (10.1105/tpc.114.134809) 25770109 PMC4558661

