# Supplementary material for: A whitefly effector Bsp9 targets host immunity regulator WRKY33 to promote performance
Source: Philos Trans R Soc Lond B Biol Sci. 2019 Jan 14;374(1767):20180313. doi: 10.1098/rstb.2018.0313 (PMC6367160; doi:10.1098/rstb.2018.0313)
Supplement: Supplementary information [file rstb20180313supp1.docx]

**SUPPLEMENTARY INFORMATION**

**A whitefly effector Bsp9 targets host immunity regulator WRKY33 to promote performance**

***Wang et al.***

**Philosophical Transactions B**

Figures S1-S6

Tables S1-S3.

Correspondence should be addressed to J.Y. (e-mail: [jianye@im.ac.cn](mailto:jianye@im.ac.cn)).

**Figure S1. Screening of whitefly salivary protein.** Flow chart of genomics screening of TYLCV induced whitefly salivary protein.

**Figure S2. Bsp9 is highly induced expression in viruliferous whitefly.** Relative expression of *Bsp9* in virus-free or viruliferous whitefly. TYLCV-viruliferous whiteflies were feed on Arabidopsis for 72 h. non-viruliferous whitefly were used as control. Values are means ± SD (n=4). (**, P< 0.01; Student’s t-test).

**Figure S3. Arabidopsis *PDF1.2* is induced by whitefly infestation.** Relative expression level of *PDF1.2*. Three-week old Col-0 plants were infested with whiteflies for indicated time. Col-0 plants without whitefly treatment was used as control. Bars represent means ± SEM (n=4). Asterisk indicates significant differences on gene expression between 0 h and other time treated (**, P< 0.01, Student’s t-test).

**Figure S4. Bsp9 secretes into plant during whitefly feeding.** (*a*) Signal peptide prediction of Bsp9 protein by SignalP4.1. (*b*) Transmembrane region prediction of Bsp9 protein by TMHMM. (*c*) Subcellular localization of Bsp9-YFP in *N.benthamiana* leaves.


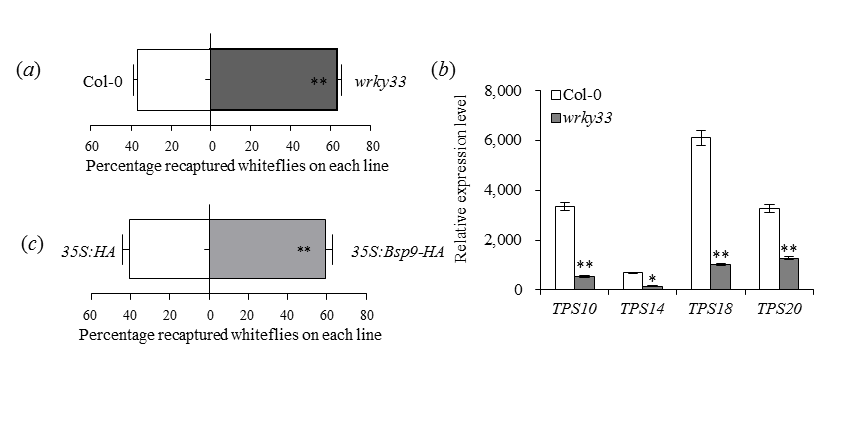


**Figure S5. Bsp9 increases whitefly preference might by interfering WRKY33.** (*a*) Whitefly preference (as percentage recaptured whiteflies out of 200 released) on *wrky33* plants compared to Col-0 plants. Values are means + SD (n=8). (*b*) Relative expression levels of Arabidopsis *Terpene Synthase genes* in Col-0 and *wrky33* mutant plants. Bars represent means ± SD (n=4). (*c*) Whitefly preference (as percentage recaptured whiteflies out of 200 released) on *35S:Bsp9-HA* plants compared to *35S:HA* transgenic Arabidopsis plants. Values are means + SD (n=8).

**Figure S6. No viral transcription is detected after viruliferous whitefly infestation for 72 h.** Relative TYLCV virus titer of Col-0 plants infested with virus-free whitefly or viruliferous whitefly for 72 h in DNA and RNA levels. Bars represent means ± SD (n=4). Asterisk indicates significant differences on TYLCV amount between virus-free whitefly and viruliferous whitefly infested plants (**, P< 0.01, Student’s t-test). The ‘ns’ indicates no significant differences.

**Table S1.** Primers sequences used in this investigation.

| **Gene** | **Primer Sequence (5’-3’)** | **Purpose** |
| --- | --- | --- |
| Bsp1-BamH1-F | GGATCCGAATTGATGGAAAAGCTTTG | Cloning |
| Bsp1-XholI-R | CTCGAGGTGTAGCACTTCAAATCACC | Cloning |
| Bsp2-BamH1-F | GGATCCGAATCATGTGTCAGCCCC | Cloning |
| Bsp2-XholI-R | CTCGAGGTCTGAGCGAGCAACTTCGA | Cloning |
| Bsp3-BamH1-F | CAAGGGATCCATGCGCTTTGAATATAAATATAG | Cloning |
| Bsp3- XholI -R | CAAGTGCTCGAGGCTCGGCGCCGGGCAGCCGG | Cloning |
| Bsp4-BamH1-F | GGATCCCGGTCTCCTAGTTCCGC | Cloning |
| Bsp4-EcoR1-R | GAATTCGCTGTGAAATTTGCGTAC | Cloning |
| Bsp5-BamH1-F | GGATCCATGCCTACATCGTTGGAGGC | Cloning |
| Bsp5-Xhol1-R | CTCGAGGTGTGGGCACAATAGTTCTTGG | Cloning |
| Bsp6-BamH1-F | GGATCCCATCCCTGCGGAGGTGGAGGCG | Cloning |
| Bsp6-EcoR1-R | GAATTCGCGAGGACGTTGTTGAGG | Cloning |
| Bsp7-BamH1-F | GGATCCGCCTTTTGGAATGTGA | Cloning |
| Bsp7-EcoR1-R | GAATTCGCGCAAGGTCCAATGGAA | Cloning |
| Bsp8-BamH1-F | GGATCCATTTCACTCAAATGTG | Cloning |
| Bsp8-EcoR1-R | GAATTCGCACACATTATTCGTCGA | Cloning |
| Bsp9-BamH1-F | CAAGGGTACCATGGGTGCTACAGAGAATCC | Cloning |
| Bsp9-EcoR1-R | CAAGTTCGAAGTTGGCCTTAAAGGAAGAGA | Cloning |
| Bsp10-BamH1-F | GGATCCGAGACATTGCTGAATGCCGA | Cloning |
| Bsp10-EcoR1-R | GAATTCGCCTTTCTACCAGGGACTACTG | Cloning |
| AtWRKY33-BamH1-F | GGATCCATGGCTGCTTCTTTTCTTAC | Cloning |
| AtWRKY33-Xhol-R | CTCGAGTG AACATCGTGGTTGTGTTTCC | Cloning |
| AD-AtWRKY33-F | CAAGGGTACCATGGCTGCTTCTTTTCTT | Y2H |
| AD-AtWRKY33-R | CAAGCTCGAGGGCATAAACGAATCGAAA | Y2H |
| BD-Bsp9-F | CAAGGAATTCATGGGTGCTACAGAGAAT | Y2H |
| BD-Bsp9-R | CAAGGTCGACGTTAGTTGGCCTTAAAGG | Y2H |
| cEYFP-Bsp9-F | CAAGGAATTCATGGGTGCTACAGAGAAT | BIFC |
| cEYFP-Bsp9-R | CAAGGGTACCCTTAGTTGGCCTTAAAGG | BIFC |
| nEYFP-AtWRKY33-F | CGGCCGAATTCATGGCTGCTTCTTTTCTTACAAT | BIFC |
| nEYFP- AtWRKY33-R | CAAGGGTACCGGGGCATAAACGAATCGAA | BIFC |
| cEYFP-AtMPK6-F | CAAGGTCGACATGGACGGTGGTTCAGGTCA | BIFC |
| cEYFP-AtMPK6-R | CAAGGGATCCCTTGCTGATATTCTGGATTGA | BIFC |
| nLUC-Bsp9-F | CAAGGGTACCATGGGTGCTACAGAGAAT | LUC assay |
| nLUC-Bsp9-R | CAAGGTCGACGTTAGTTGGCCTTAAAGG | LUC assay |
| cLUC-AtWRKY33-F | CAAGGGTACCATGGCTGCTTCTTTTCTT | LUC assay |
| cLUC-AtWRKY33-R | GAACGTCGACGGGCATAAACGAATCGAA | LUC assay |
| nLUC-AtMPK6-F | CAAGGGTACCATGGACGGTGGTTCAGGTCA | LUC assay |
| nLUC- AtMPK6-R | CAAGGTCGACTTGCTGATATTCTGGATTGA | LUC assay |
| AtPEPR1-pF | GTTTTGGCTGAGGAAAGACG | qRT-PCR |
| AtPEPR1-pR | ACATTGTACCGTGCAGACCA | qRT-PCR |
| AtPDF1.2-qF | TTTGCTGCTTTCGACGCAC | qRT-PCR |
| AtPDF1.2-qR | GATTCTTGCATGCATTACTG | qRT-PCR |
| TYLCV-qF | TATGTTAGCTATTAAATATTTGC | qRT-PCR |
| TYLCV-qR | AGCACGGCTGCTGTATGGGC | qRT-PCR |
| At-Tubulin2-qF | TCAAGAGGTTCTCAGCAGTA | qRT-PCR |
| At-Tubulin2-qR | TCACCTTCTTCATCCGCAGTT | qRT-PCR |
| BtTAF(nc)-qF | TGTGGGACACCCATTATCAG | qRT-PCR |
| BtTAF(nc)-qR | TGTGCAGCCAAGGAAATAAG | qRT-PCR |
| At-TPS10-qF | GTACATGCAAAATGCTCGGAT | qRT-PCR |
| At-TPS10-qR | TTGGTGTTGGGACAAAGTCTC | qRT-PCR |
| At-TPS14-qF | AGGCGAAGAACTAACAAAAGAG | qRT-PCR |
| At-TPS14-qR | AGAATGGACATGGATTCAGACA | qRT-PCR |
| At-TPS18-qF | ACCGAAAGGACGATCATACG | qRT-PCR |
| At-TPS18-qR | GCTGTCACCAACCCAACTTT | qRT-PCR |
| At-TPS20-qF | TGGAATAGTGACTGGGGGAG | qRT-PCR |
| At-TPS20-qR | TTCCTGTCGCAATCTCTCCT | qRT-PCR |
| Actin2-F | CGTTTCGCTTTCCTTAGTGTTAGCT | qRT-PCR |
| Actin2-R | AGCGAACGGATCTAGAGACTCACCTTG | qRT-PCR |

**Table S2.** Summary of whitefly salivary proteins Bsp1-10 in functional analysis of plant immunity activation assay

| **Name** | **Gene ID** | **Annotation** | **Function** | **GenBank number** |
| --- | --- | --- | --- | --- |
| Bsp1 | c499 | Unknown | Activate | XM_019056712.1 |
| Bsp2 | c1701 | translocon-associated | Unchange | XM_019060030.1 |
| Bsp3 | c4622 | Lectin | Inhibit | XM_019059181.1 |
| Bsp4 | c43647 | Unknown | Unchange | MAMS01017023.1 |
| Bsp5 | c43964 | Unknown | Activate | MAMS01012701.1 |
| Bsp6 | c45278 | Unknown | Unchange | XM_001993075.1 |
| Bsp7 | c51075 | Unknown | Inhibit | XM_019041733.1 |
| Bsp8 | c55919 | Unknown | Inhibit | XM_019053341.1 |
| Bsp9 | c57561 | Unknown | Inhibit | KY986869.1 |
| Bsp10 | c69151 | Unknown | Unchange | XM_019046870.1 |

**Table S3** Genomics analysis of Bsp9 in two invasive species of MEAM1 and MED whiteflies
